# An interactive visualization tool for educational outreach in protein contact map overlap analysis

**DOI:** 10.3389/fbinf.2024.1358550

**Published:** 2024-03-15

**Authors:** Kevan Baker, Nathaniel Hughes, Sutanu Bhattacharya

**Affiliations:** ^1^ Department of Computer Science and Software Engineering, Auburn University, Auburn, AL, United States; ^2^ Department of Computer Science and Computer Information Systems, Auburn University at Montgomery, Montgomery, AL, United States

**Keywords:** protein structure, contact map, contact map overlap, template based modeling, protein structure prediction

## Abstract

Recent advancements in contact map-based protein three-dimensional (3D) structure prediction have been driven by the evolution of deep learning algorithms. However, the gap in accessible software tools for novices in this domain remains a significant challenge. This study introduces GoFold, a novel, standalone graphical user interface (GUI) designed for beginners to perform contact map overlap (CMO) problems for better template selection. Unlike existing tools that cater more to research needs or assume foundational knowledge, GoFold offers an intuitive, user-friendly platform with comprehensive tutorials. It stands out in its ability to visually represent the CMO problem, allowing users to input proteins in various formats and explore the CMO problem. The educational value of GoFold is demonstrated through benchmarking against the state-of-the-art contact map overlap method, map_align, using two datasets: PSICOV and CAMEO. GoFold exhibits superior performance in terms of TM-score and Z-score metrics across diverse qualities of contact maps and target difficulties. Notably, GoFold runs efficiently on personal computers without any third-party dependencies, thereby making it accessible to the general public for promoting citizen science. The tool is freely available for download for macOS, Linux, and Windows.^1^

## 1 Introduction

The study of protein three-dimensional (3D) structure prediction has undergone rapid development in recent years, driven largely by advancements in accurate prediction of inter-residue contact map powered by deep learning algorithms (Abriata et al., 2019; Hou et al., 2019; Senior et al., 2019; Xu and Wang, 2019; AlQuraishi, 2021). Despite these strides, the field still faces a significant challenge: the dearth of software tools accessible to novices. Existing tools often fail to bridge the gap between advanced protein folding methodologies and foundational learning for beginners (McGehee et al., 2020). Historically, tools like FoldIt (Kleffner et al., 2017) have been instrumental in demystifying protein folding for the lay audience. FoldIt, an online platform, leverages the power of crowdsourcing and the Rosetta molecular modeling software (Leaver-Fay et al., 2011; Adolf-et al., 2013; Schenkelberg and Bystroff, 2015), allowing users to engage in solving protein folding puzzles. While FoldIt and its standalone version address aspects of protein folding, they primarily cater to research needs and do not offer comprehensive guidance for beginners. Similarly, PolyFold (McGehee et al., 2020) provides user-friendly manipulation of protein structures but falls short in offering the level of assistance that beginners might require https://drive.google.com/drive/folders/1_hQ5Yy0seCdfC71KMzRBqMM1pIM0uNJE?usp=sharing.

Moreover, protein 3D structure prediction, including template-based methods (Yang et al., 2011; Ma et al., 2012; Bhattacharya and Bhattacharya, 2019a; Bhattacharya and Bhattacharya, 2019b; Xu and Wang, 2019; Bhattacharya and Bhattacharya, 2020; Zhang and Shen, 2020; Bhattacharya, 2021; Bhattacharya, 2021; Bhattacharya et al., 2022; Bhattacharya et al., 2023), has been revolutionized by the use of contact maps powered by deep learning. A contact map represents the three-dimensional (3D) structure of a protein by capturing the spatial closeness between residues. These maps have been shown to considerably enhance the accuracy of template-based protein 3D structure prediction (Hou et al., 2019; Xu and Wang, 2019; Pearce and Zhang, 2021). In this context, the analysis of contact map overlap (CMO) becomes vital for evaluating the suitability of a template protein in template-based modeling (Ovchinnikov et al., 2017; Bhattacharya and Bhattacharya, 2019b; Bhattacharya et al., 2022). Researchers have addressed the CMO problem with different approaches (Di Lena et al., 2010; Buchan and Jones, 2017; Bhattacharya et al., 2022). In particular, Al-Eigen (Di Lena et al., 2010), EigenTHREADER (Buchan and Jones, 2017), and CEthreader (Zheng et al., 2019) are tools which calculate the eigenvectors of the contact maps for two input proteins, and compare them by performing a global alignment of the eigenvectors by utilizing eigendecomposition. GR-Align (Malod-Dognin and Pržulj, 2014), a program which analyzes the two proteins in the CMO problem as graphs and graphlets, is intended for large-scale testing of databases for alignments. map_align (Ovchinnikov et al., 2017), inspired by (Taylor, 1999), analyzes the target and template proteins using scoring matrices determined by initially using the Smith-Waterman (Smith and Waterman, 1981) algorithm to identify subsequence alignments and determine the best alignment between the target and template proteins. However, these tools do not allow the visualization of the contact map overlapping and some tools (Ovchinnikov et al., 2017; Zheng et al., 2019; Bhattacharya et al., 2022) take sequential and structural features along with contact information to calculate the CMO score, making them time consuming (Bhattacharya et al., 2022) and difficult to use for novice.

GoFold–our novel, standalone GUI design to demystify the basics of protein 3D structure prediction and CMO problem. Unlike tools that assume a foundational understanding of the subject, GoFold is tailored for beginners. It features a user-friendly interface with collapsible, accessible tutorials that guide users through the basic functionalities of protein structure prediction. This approach allows novices to grasp the complexities of the field without the added layer of confusion. GoFold stands out in its ability to visualize the CMO problem. While existing tools offer unique methodologies for analyzing protein alignments, they do not provide a visual representation of the overlap. GoFold addresses this gap by allowing users to input proteins in either the CASP residue-residue format (Senior et al., 2019; Kryshtafovych et al., 2021) or the common PDB format (Berman et al., 2000), offering a visual display of the distance maps and their overlaps. The tool is freely available for download at https://drive.google.com/drive/folders/1_hQ5Yy0seCdfC71KMzRBqMM1pIM0uNJE?usp=sharing for macOS, Linux, and Windows.

## 2 Methods and materials

### 2.1 GoFold’s features

**FIGURE 1:**
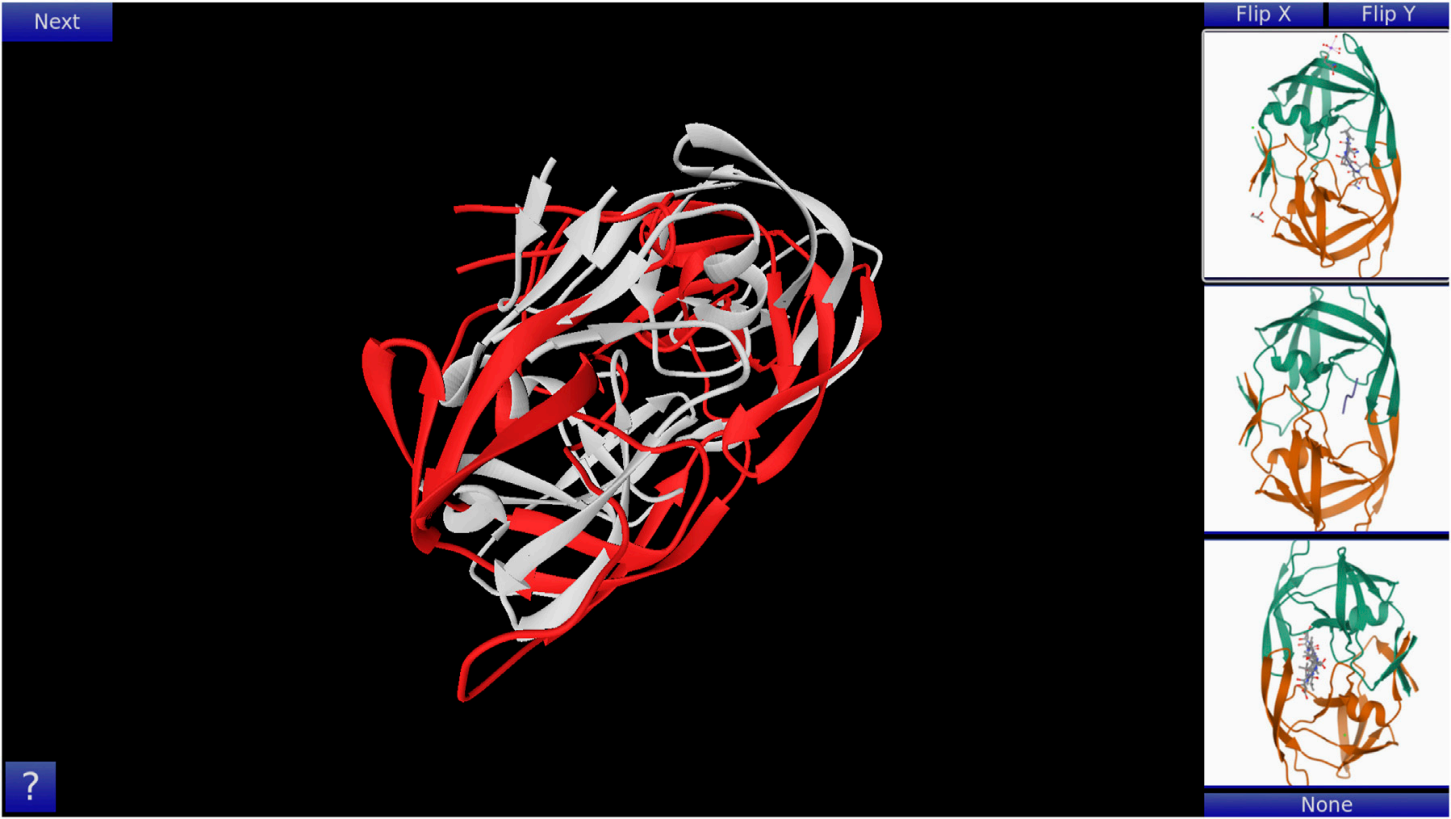
A representative Template Matching mode of GoFold. A template (in white) is selected, which is overlayed across the target (in red).

**FIGURE 2:**
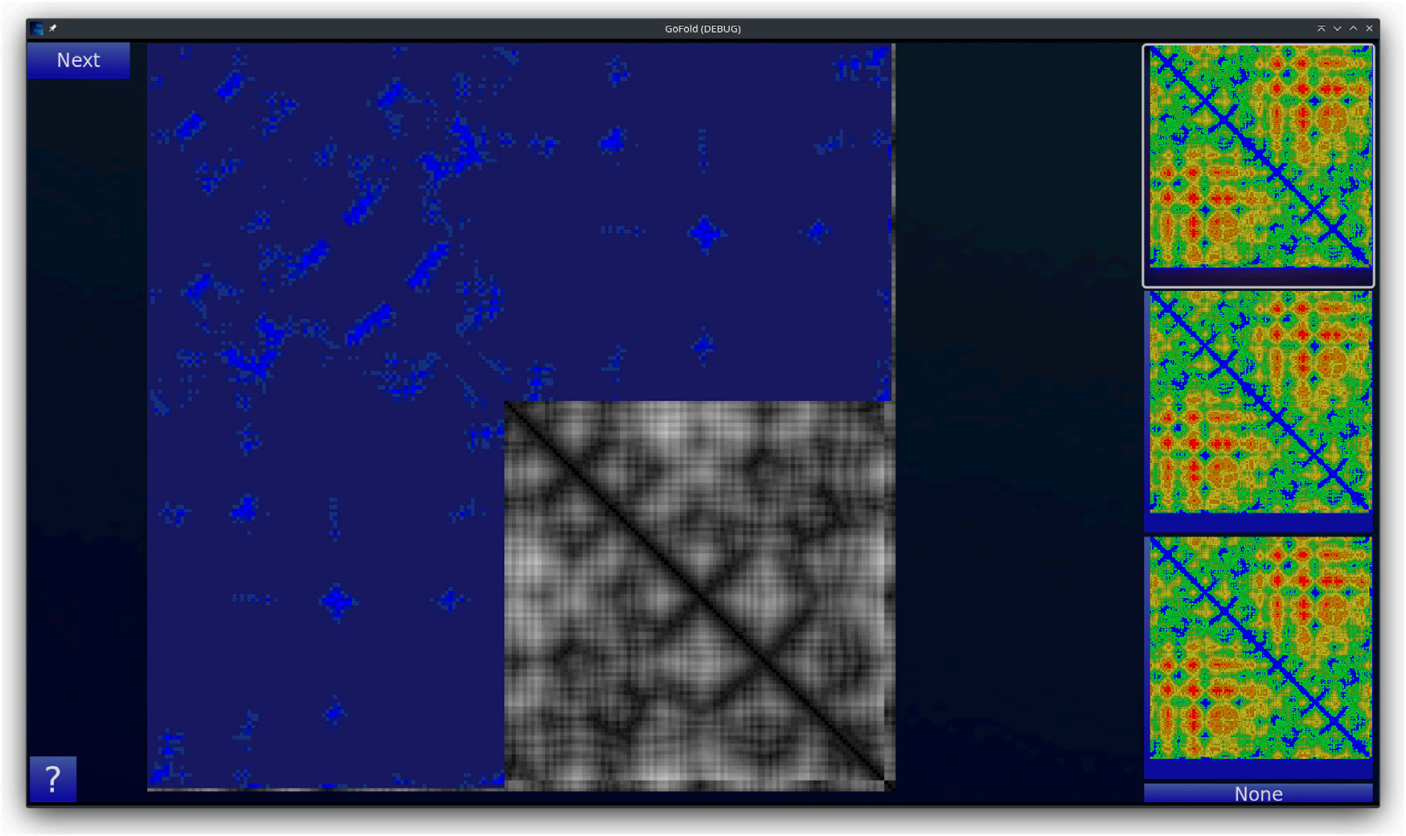
A representative Contact Map Matching mode of GoFold. A template contact map is selected and is overlayed (in blue) across the target contact map (in gray).

GoFold is designed to simulate and educate users about the intricacies of protein folding. The primary components of GoFold are divided within two basic modes, each crafted to offer a unique aspect of protein folding. *Template Matching Mode*: In the Template Matching Mode, users are introduced to template-based protein folding through an interactive 3D interface. Initially, 3D structures, prepared in Chimera (Pettersen et al., 2004), are imported into GoFold, allowing users to manually manipulate these structures to match the template closely with the target protein. This hands-on approach aids users in visually identifying the most suitable template for their target protein. As shown in Figure 1, the interface displays a 3D structure of a target protein shaded in red. The core challenge for the user is to select the best-fit template for the given target. Adjacent to the target, on the right side of the interface, three template options are presented as clickable buttons. When a template is selected, it is rendered in white and positioned at the same origin point as the target protein for direct comparison (refer to Figure 1). This visual juxtaposition allows the user to evaluate and choose the template that best matches the target. Additionally, users can adjust the camera angle via mouse controls, enabling a comprehensive view of the protein structures. Importantly, users can only select one template at a time, mirroring the critical decision-making process in template-based protein folding. *Contact Map Matching Mode*: Diverging from the 3D visualization, this mode presents the user with two-dimensional (2D) contact (or distance) maps of target and template proteins. As shown in Figure 2, the user selects a template contact map from the right-hand side of the screen. Upon selection, the template is superimposed on the target contact map with the target contact map shifting to grayscale and the template map adopting a blue hue (refer to Figure 2). The objective remains consistent with the previous mode. This mode emphasizes understanding spatial relationships and distances within protein structures. The next subsection outlines the approach taken by GoFold to address the contact map overlap problem.

### 2.2 GoFold’s contact map overlap approach

In addressing the contact map overlap problem, our approach employs a novel strategy inspired by previous studies (Taylor, 1999; Ovchinnikov et al., 2017; Bhattacharya et al., 2022). Our algorithm is designed to enhance the accuracy of contact map alignment through a two-step dynamic programming process (Supplementary Text S1). *First Step:* In this phase, we calculate scores for each row (representing a specific residue) of the first contact map against each row of the second contact map. The score computation involves the summation of Gaussian functions: exp {-x^2^/[2y (Xu and Wang, 2019)]}, where “x” is the difference in sequence separation of aligned contacts, and “y” (standard deviation) is a function of the smaller of the two sequence separations. Dynamic programming is then employed to identify the alignment of contacts for the two rows that maximizes the sum of these Gaussian functions. The optimized scores are recorded in a second matrix. *Second Step:* To refine the alignment, we utilize the Smith–Waterman algorithm in a second dynamic programming phase. This process iterates once, updating the second-step similarity matrix based on the current alignment. This iterative refinement addresses the overestimation issue in individual row-row comparisons encountered in the first step.

The integration of this two-step dynamic programming process into GoFold’s Contact Map Matching Mode not only enhances the game’s educational value but also mirrors the complexities encountered in real-world protein folding scenarios. By simulating these intricate processes, GoFold offers an immersive learning experience, enabling users to grasp the introductory concept of protein structure alignment and analysis.

It is important to note the adaptability of GoFold to different user needs. For Figure 1, the 3D structures are pre-generated and imported, allowing users to interactively explore template matching through manual adjustments. In contrast, the contact maps shown in Figure 2 are outcomes of GoFold’s algorithm, utilized in an educational context to simplify the concept for users. To accommodate a range of experiences, from novices to advanced users, GoFold’s design intentionally avoids real-time computation in the Contact Map Matching Mode for enhanced user experience and accessibility. Advanced users, however, have the option in a subsequent phase to directly input contact maps and engage with the CMO algorithm for a comprehensive exploration of GoFold’s capabilities.

### 2.3 Benchmarking datasets, competing methods, and evaluation metrics

While GoFold is primarily an interactive visual simulator for protein folding as opposed to a protein structure prediction method, we assess GoFold’s contact map overlap predictive ability using two benchmarking datasets. Our first benchmark dataset is the PSICOV dataset (Jones et al., 2012), containing 116 single chain, single domain proteins with a length cutoff of 200 to focus our analysis on small target proteins. In order to test the impact of different types of contact maps in the performance, we consider predicted contact maps including (i) sparse inverse covariance estimation method (PSICOV) (Jones et al., 2012), (ii) state-of-the-art deep learning method (trRosetta) (Yang et al., 2020), and (iii) native (experimentally determined) contact maps. PSICOV contacts are collected from the MetaPSICOV benchmark dataset (Jones et al., 2015). trRosetta contact maps are collected by submitting jobs to the trRosetta server (https://yanglab.nankai.edu.cn/trRosetta/).

Our second benchmark dataset is the CAMEO dataset (Haas et al., 2019) officially released from 20 August 2022 to 11 February 2023 with a length ranges from 50 to 150. This dataset contains 25 easy, 39 medium, and 16 hard targets. On this dataset, we use predicted contact maps by trRosetta by submitting jobs to the trRosetta server. These maps are predicted as intermediate features by trRosetta, alongside other features, while predicting 3D models. We utilize these intermediate contact maps directly and do not derive contact information from the final trRosetta-predicted 3D structures.

We use a representative non-redundant library of templates, collected from: https://zhanglab.ccmb.med.umich.edu/library/, (Roy et al., 2010; Yang et al., 2015). We use TM-align (Zhang and Skolnick, 2005) to randomly select template(s) for each target with a TM-score <0.9 to the target, avoiding the chance of selecting the native structure as a template for a target. Following this procedure, we have 184 target-template pairs for the PSICOV dataset, and 46 easy, 39 medium and 30 hard target-template pairs for the CAMEO dataset. Recognizing that medium and hard targets are often challenging to model with a single template, our strategy in selecting templates is driven by the understanding that even partial alignments with medium and hard targets can provide valuable insights into protein structure prediction.

Over these datasets, the performance of GoFold is benchmarked against the state-of-the-art contact map overlap method, map_align (Ovchinnikov et al., 2017). To run map_align and GoFold, we only use contact maps as inputs. We cannot include recent contact map overlap methods (Di Lena et al., 2010; Buchan and Jones, 2017; Zheng et al., 2019; Bhattacharya et al., 2022) for benchmarking because either the tool is not publicly available, or it requires other sequential and/or structural features along with contact maps as inputs. Here, it is worth mentioning that GoFold is specifically designed to work efficiently with just contact maps, streamlining its use for educational purposes and making it highly accessible to users without the need for generating other sequential and structural features.

The predicted target-template alignment quality by GoFold and map_align is evaluated using the Z-score by CCpro (Di Lena and Baldi, 2021). CCpro outputs a Z-score, the greater the value the better the alignment. In addition, the output alignments are then fed into MODELLER (Webb et al., 2014) to build the 3D structures of the target proteins. TM-score (Xu and Zhang, 2010) is used to evaluate the quality of the predicted 3D structure of target proteins with respect to the native (experimentally determined) structures. The value of TM-score lies in the range (0,1), where a higher score indicates better similarity. A TM-score >0.5 indicates a correct fold to the native structure. To make a fair comparison, the same contact maps, the same template, and the same modeling strategy by MODELLER are used for both competing methods.

## 3 Results and discussion

### 3.1 Performance on PSICOV dataset with contact maps of diverse qualities

To investigate the impact of quality of contacts on the performance, we benchmark our method, GoFold, against the state-of-the-art contact map overlap method, map_align, using contact maps of diverse qualities on the PSICOV dataset. Notably, we include contact maps predicted by PSICOV, trRosetta, and native (true) contact maps. To make a fair comparison, the same contact maps, the same template and the same modeling strategy by MODELLER are used for both competing methods.

*Using PSICOV Predicted Contact Maps:* As shown in Table 1, GoFold exhibits superior performance using PSICOV predicted contact maps with a mean TM-score of 0.4297 and a mean Z-score of 25.9258, compared to map_align with a mean TM-score of 0.4053 and a mean Z-score of 22.8816. The performance improvement of GoFold over map_align is also statistically significant at the 95% confidence level (*p* < 0.05). We also note that GoFold outperforms map_align by accurately predicting the correct fold with a TM-score exceeding 0.5 in 64 out of 184 target-template pairs, surpassing map_align’s performance of 55 pairs. *Using trRosetta Predicted Contact Maps:* Moreover, GoFold demonstrates a statistically significant advantage over map_align when employing high-quality contacts from trRosetta. GoFold achieves a mean TM-score of 0.5307 compared to map_align’s 0.4891, showcasing GoFold’s ability to surpass a mean TM-score of 0.5 and map_align falls short to achieve a mean TM-score of 0.5. Furthermore, GoFold outperforms map_align by accurately predicting the correct fold (TM-score >0.5) in 93 out of 184 target-template pairs as opposed to 79 by map_align. The Z-scores also affirm the robust performance of GoFold over map_align (52.732 vs. 44.466). *Using Native Contact Maps:* GoFold continues to exhibit a statistically significant superiority over map_align when utilizing native contact maps, showcasing a substantial margin in both mean TM-score (∼0.06) and mean Z-score (∼11). In terms of predicting the correct fold, GoFold achieves correct folds in 107 out of 184 pairs compared to map_align’s 84, illustrating while GoFold predicts models with TM-score >0.5 for 23 cases, map_align falls short to achieve it.

**TABLE 1:** Performance comparison on PSICOV dataset based on the mean TM-score of predicted models and mean Z-score of target-template alignments. One sample *t*-test’s *p*-value is shown in brackets. We include contact maps predicted by PSICOV, trRosetta, and true (native) contact maps. Best performance are listed in bold.

Contact source	Mean TM-score		Mean Z-score	
	map_align (*p-value*)	GoFold	map_align (*p-value*)	GoFold
PSICOV	0.4053 (*1.9495E-06*)	**0.4297**	22.8816 (*9.8636E-13*)	**25.9258**
trRosetta	0.4891 (*5.0218E-17*)	**0.5307**	44.4660 (*1.4591E-25*)	**52.7320**
Native Contact	0.5083 (*6.3859E-20*)	**0.5607**	47.8723 (*8.0532E-29*)	**58.1173**

**FIGURE 3:**
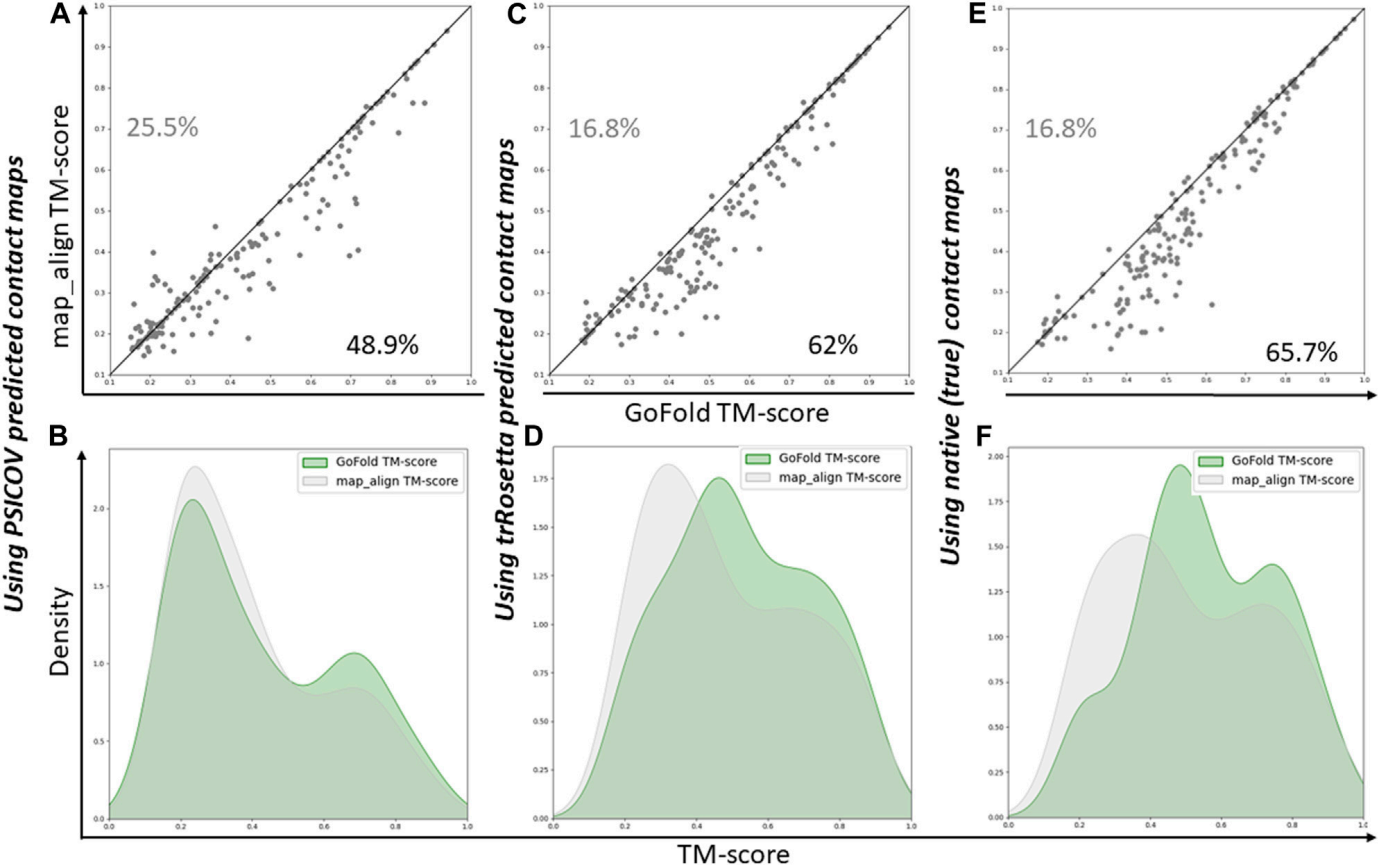
A head-to-head Performance comparison of GoFold and map_align on PSICOV dataset based on the TM-score of predicted models. We include contact maps predicted by PSICOV, trRosetta, and true (native) contact maps. **(A)** GoFold versus map_align using PSICOV predicted contact maps, **(B)** TM-score distribution of models predicted by GoFold (in green) versus map_align (in grey) using PSICOV predicted contact maps, **(C)** GoFold versus map_align using trRosetta predicted contact maps, **(D)** TM-score distribution of models predicted by GoFold (in green) versus map_align (in grey) using trRosetta predicted contact maps, **(E)** GoFold versus map_align using native (or true) contact maps, **(F)** TM-score distribution of models predicted by GoFold (in green) versus map_align (in grey) using native (or true) contact maps.

As shown in Figure 3A, GoFold outperforms map_align in 48.9% of cases, emphasizing GoFold’s resilience with low-quality PSICOV predicted contacts. We see a similar trend in Figure 3B where the distribution of GoFold is towards the higher TM-score than that of map_align, illustrating that GoFold predicts models with a higher TM-score than that of map_align. Figure 3C further illustrates that GoFold, using high-quality trRosetta predicted contact maps, outperforms map_align using the same contact maps for 62% of the cases, whereas 16.8% of the cases show that map_align outperforms GoFold. Using the same contact maps, we observe a similar trend when we plot the distribution plot (Figure 3D) of predicted models by GoFold and map_align. Figures 3E, F further demonstrate the superior performance of GoFold over map_align using native contact maps. In particular, while GoFold outperforms map_align in 65.7% of the cases, map_align outperforms GoFold in 16.5% of the cases. Moreover, as shown in Figure 3E, the peak of GoFold’s distribution plot is higher as well as towards the higher TM-score range than map_align, illustrating that GoFold significantly outperforms map_align. Additionally, when evaluating the target-template alignment prediction quality in terms of Z-score (Supplementary Figure S1), a similar trend is observed, further highlighting GoFold’s superiority over map_align in terms of Z-score of predicted alignments. Overall, we note the impact of different qualities of contacts on the performance of GoFold and map_align, as well as the statistically significantly superior performance of GoFold over map_align in terms of predicting the on the PSICOV dataset.

### 3.2 Performance on CAMEO dataset

The performance of GoFold is further benchmarked against map_align on the CAMEO dataset, containing 25 easy targets (results into 46 target-template pairs), 39 medium targets (results into 78 target-template pairs), and 16 hard targets (results into 30 target-template pairs). The target difficulty is officially assigned by CAMEO.

The comparative evaluation of predictive performance, as measured by TM-score and Z-score metrics, between map_align and GoFold across different target categories is presented in Table 2. Notably, GoFold consistently demonstrates superior predictive capabilities across all target difficulty categories. For easy targets, GoFold achieves a TM-score of 0.4879, surpassing map_align’s TM-score of 0.4260, with a *p*-value of 1.5913E-05. Moreover, while GoFold predicts the correct fold with a TM-score>0.5 for 23 (out of 46) cases, map_align only predicts it for 14 cases. In medium difficulty targets, GoFold achieves a TM-score of 0.4402, outperforming map_align’s 0.4058 with a *p*-value of 1.8842E-05. In terms of correct folds prediction, GoFold achieves TM-score>0.5 for 27 cases, which is 6 more than that of map_align. On hard targets, GoFold achieves a TM-Score of 0.3565, surpassing map_align’s 0.3254, with a *p*-value of 0.04666. Overall, across all targets, GoFold yields a superior mean TM-Score of 0.4381 compared to map_align’s 0.3962, with a statistically significant *p*-value of 2.4115E-19. Moreover, GoFold outperforms map_align in terms of predicting the correct folds with TM-score>0.5 (7 vs. 6), illustrating the advantage of GoFold across all target difficulty over map_align. Similar trends are observed when considering Z-Scores, with GoFold consistently outperforming map_align across all target categories, emphasizing the statistically significantly superior performance of GoFold over map_align in predicting higher-quality models and alignments.

**TABLE 2:** Performance comparison of GoFold against map_align on CAMEO dataset based on the mean TM-score of predicted models and mean Z-score of target-template alignments. One sample *t*-test’s *p*-value is shown in brackets. The target category is officially released by CAMEO. Best performance are listed in bold.

Evaluation metrics	Target category	map_align	GoFold
Mean TM-score	Easy	0.4260 (*1.5913E-05*)	**0.4879**
	Medium	0.4058 (*1.8842E-05*)	**0.4402**
	Hard	0.3254 (*0.04666*)	**0.3565**
	All	0.3962 (*2.4115E-19*)	**0.4381**
Mean Z-score	Easy	36.9084 (*2.5487E-08*)	**44.8056**
	Medium	36.0894 (*6.123E-08*)	**42.6156**
	Hard	33.2852 (*2.2119E-06*)	**40.1224**
	All	35.7877 (*7.8818E-19*)	**42.7841**

**FIGURE 4:**
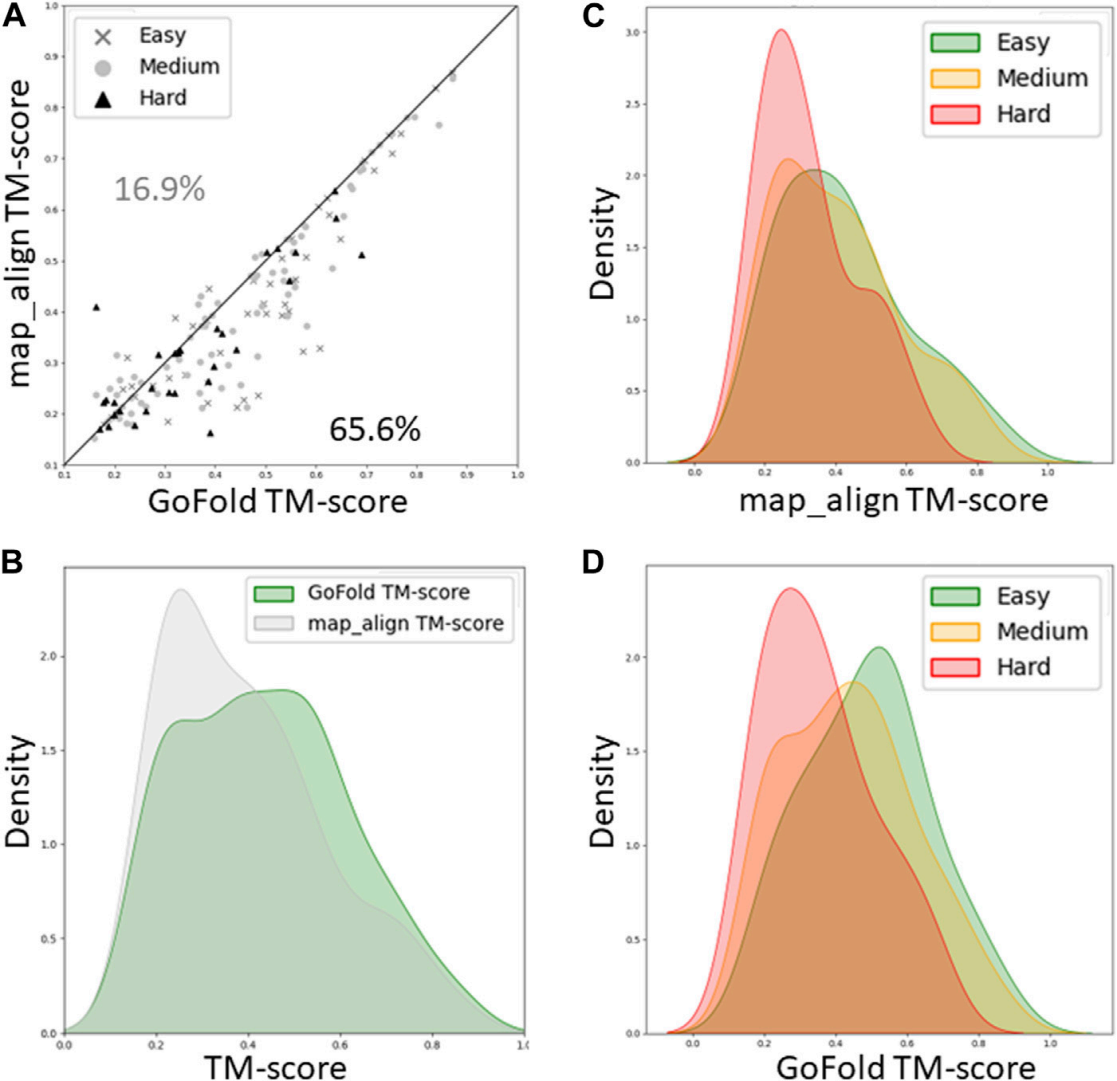
A head-to-head Performance comparison of GoFold and map_align on CAMEO dataset based on the TM-score of predicted models. The target category is officially released by CAMEO. We include contact maps predicted by trRosetta for both GoFold and map_align. **(A)** GoFold versus map_align, **(B)** TM-score distribution of models predicted by GoFold (in green) versus map_align (in grey) over all targets, **(C)** TM-score distribution of models predicted by map_align over easy (in green), medium (in yellow), hard (in red) targets. **(D)** TM-score distribution of models predicted by GoFold over easy (in green), medium (in yellow), hard (in red) targets.

As shown in Figure 4A, while GoFold outperforms map_align in 65.6% of cases (out of 154), map_align is better only for 16.9% of cases, illustrating superior performance of GoFold in predicting higher TM-score than that of map_align. Figure 4B shows the TM-score distribution for GoFold is towards higher TM_score range compared to map_align, demonstrating GoFold predicts more models with higher TM-score than that of map_align. Figures 4C, D further show the TM-score distribution of predicted models by map_align and GoFold, respectively, for different target categories, demonstrating the superior performance of GoFold over map_align across all target categories. Moreover, we note a similar trend when evaluating the prediction quality in terms of Z-score (Supplementary Figure S2), illustrating GoFold’s superiority over map_align in terms of Z-score of predicted alignments across all target categories.

### 3.3 Case study

As a representative example, we present a case study on CAMEO hetero-oligomer target: 7xhsA of 104 residues. As per the CAMEO official target classification, 7xhsA is categorized as hard. To ensure a fair comparison between GoFold and map_align, the same contact maps predicted by trRosetta, the same template and the same modeling strategy by MODELLER are used for both competing methods. Moreover, the recent CASP (Senior et al., 2019) experiments highlighted exceptional ability of AlphaFold2 (Senior et al., 2019; Jumper et al., 2021) in predicting protein 3D structures, significantly outperforming other groups. AlphaFold2, an end-to-end deep learning-based protein structure prediction method, utilizes a variety of sequential and structural features, including distance maps, which are integral to its superior performance. Given AlphaFold2’s advanced methodology, a direct comparison with an educational tool such as GoFold, which is designed primarily for educational purposes and uses a simpler input feature, may not be entirely fair. Nonetheless, we include AlphaFold2’s performance on the target 7xhsA. Using the Colab notebook (Mirdita et al., 2022) with default parameter settings, AlphaFold2 achieved a TM-score of 0.562, demonstrating the effectiveness of its deep learning approach and the importance of comprehensive feature utilization in predicting high-quality protein 3D structures.

**FIGURE 5:**
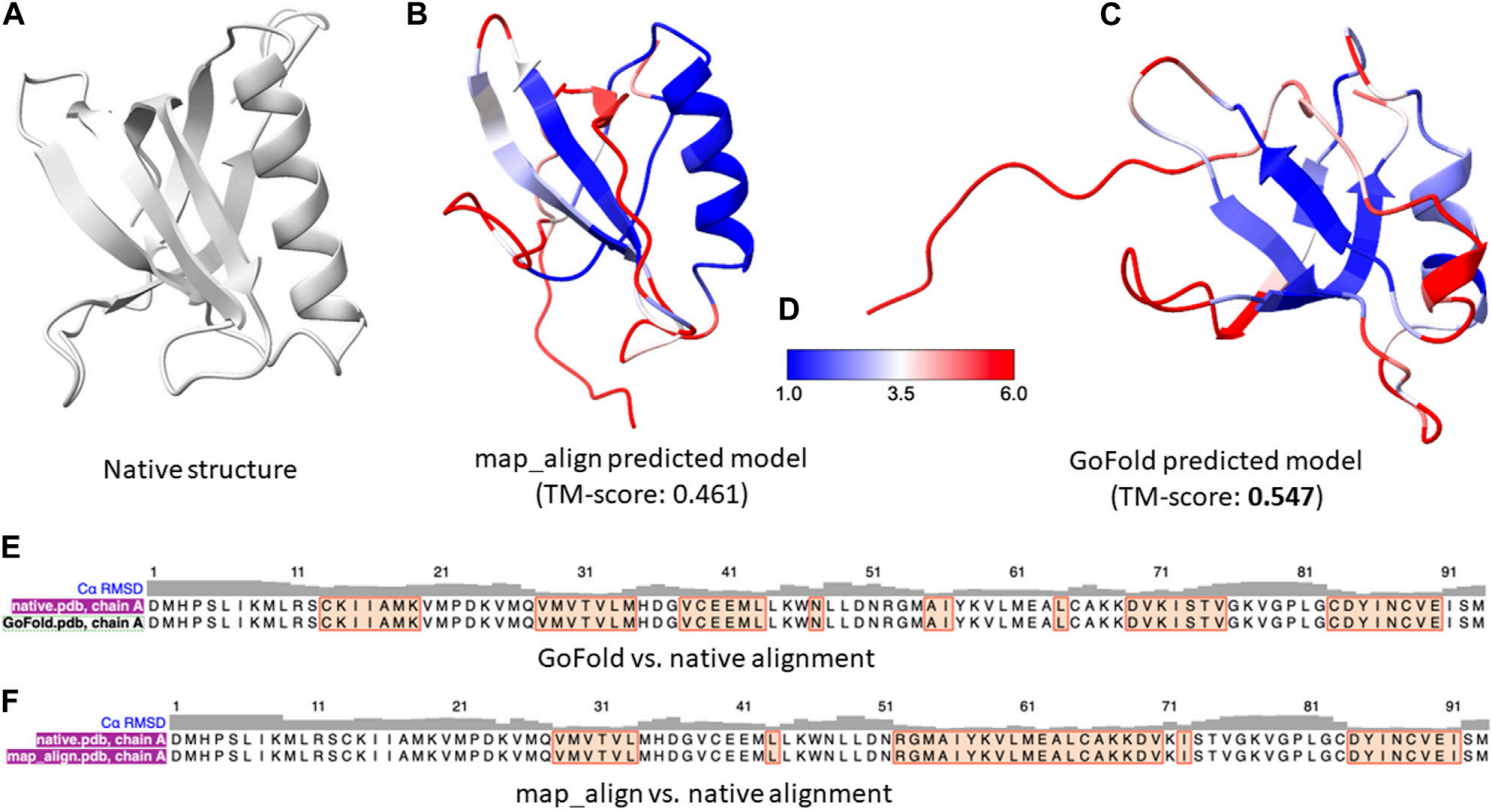
A representative example on CAMEO target 7xhsA. The target is officially classified as hard by CAMEO. **(A)** Native 3D structure of 7xhsA. **(B)** Predicted model by map_align with a TM-score of 0.461. **(C)** Predicted model by GoFold with a TM-score of 0.547. **(D)** Color bar **(E)** The sequence alignment between GoFold and native structure. **(F)** The sequence alignment between map_align and native structure.

In contrast, despite the inherent limitations when compared to the state-of-the-art predictive method, AlphaFold2, as shown in Figures 5B, C, GoFold predicts the correct fold with a TM-score of 0.547 (and a Z-score of 30.038), whereas map_align predicts an incorrect fold with a TM-score of 0.4849 (and a Z-score of 23.831), demonstrating GoFold’s ability over map_align in predicting the correct fold with TM-score >0.5 as well as high-quality target-template alignments (measured by Z-score).

## 4 Conclusion

GoFold represents a significant stride in bridging the gap between advanced protein folding methodologies and foundational learning for beginners. Its user-friendly interface and comprehensive tutorials demystify the complexities of protein 3D structure prediction, making it a valuable tool for both educational and research purposes. Benchmarking results from the PSICOV and CAMEO datasets clearly demonstrate GoFold’s superior predictive capabilities over existing method, map_align, particularly in handling diverse contact map qualities and various target difficulties. The success of GoFold in various test scenarios underscores its potential as a crucial educational and research tool in the rapidly evolving field of protein structure prediction.

## Data Availability

The original contributions presented in the study are included in the article/Supplementary Material, further inquiries can be directed to the corresponding author.
