# Supplementary material for: An interactive visualization tool for educational outreach in protein contact map overlap analysis
Source: Front Bioinform. 2024 Mar 15;4:1358550. doi: 10.3389/fbinf.2024.1358550 (PMC10982686; doi:10.3389/fbinf.2024.1358550)
Supplement: Supplementary file 1 [file DataSheet2.PDF]

Supplementary Material

for

An Interactive Visualization Tool for Educational Outreach in Protein Contact Map Overlap Analysis

Kevan Baker[1], Nathaniel Hughes[1], Sutanu Bhattacharya[1*]

[1]Department of Computer Science and Computer Information Systems, Auburn University at Montgomery, Montgomery, Alabama, USA

*** Correspondence:**
Corresponding Author

Supplementary Text 1. The pseudocode for calculating inter-residue contact maps alignment:

```
for separation_x in (0, 1, 2)
    for separation_y in (1, 2, 4, 8, 16, 32)
        initial_contact_map = setup_contact_map (separation_x, separation_y)
        for gap_penalty in (-0.2, -0.1, -0.01, -0.001)
            contact_map = initial_contact_map
            alignment_result = calculate_alignment(contact_map, -1, gap_penalty)
            alignment_score = calculate_SW_score(contact_map, -1, -0.01) / 2
            if (alignment_score > highest_score)
                optimal_alignment = alignment_result
                highest_score = alignment_score
        display(optimal_alignment)


function setup_contact_map(x_sep, y_sep)
    for col_a in columns_of_map_a
        for col_b in columns_of_map_b
            for value_a in col_a
                for value_b in col_b
                    delta_a = col_a - value_a
                    delta_b = col_b - value_b
                    if (delta_a > 0 and delta_b > 0) or (delta_a < 0 and delta_b < 0)
                        difference = abs(abs(delta_a) - abs(delta_b))
                        min_sep = min(abs(delta_a), abs(delta_b))
                        standard_dev = y_sep * (1 + (min_sep - 2) ^ x_sep)
                        weight = separation_weight(min_sep) * apply_gaussian(0, standard_dev, difference)
```

```
            contact_map[value_a][value_b] = map_a[col_a][value_a] * map_b[col_b][value_b] * weight
          else
            contact_map[value_a][value_b] = -1
  contact_map[col_a][col_b] = calculate_SW(contact_map, 0, 0)
  return contact_map


function calculate_alignment(contact_map, open_gap, extend_gap)
    alignment_map = calculate_SW(contact_map, open_gap, extend_gap)
    for col_a in columns_of_map_a
      for col_b in columns_of_map_b
        alignment_score = 0
        for value_a in col_a
          value_b = alignment_map[value_a]
          delta_a = col_a - value_a
          delta_b = col_b - value_b
          if (delta_a > 0 and delta_b > 0) or (delta_a < 0 and delta_b < 0)
            weight = separation_weight(min(abs(delta_a), abs(delta_b)))
            alignment_score += map_a[col_a][value_a] * map_b[col_b][value_b] * weight
  return alignment_map


function separation_weight(sequence_separation)
      return 1.00


function calculate_SW(matrix, open_gap, extend_gap)
  return local_alignment_score using Smith-Waterman
```

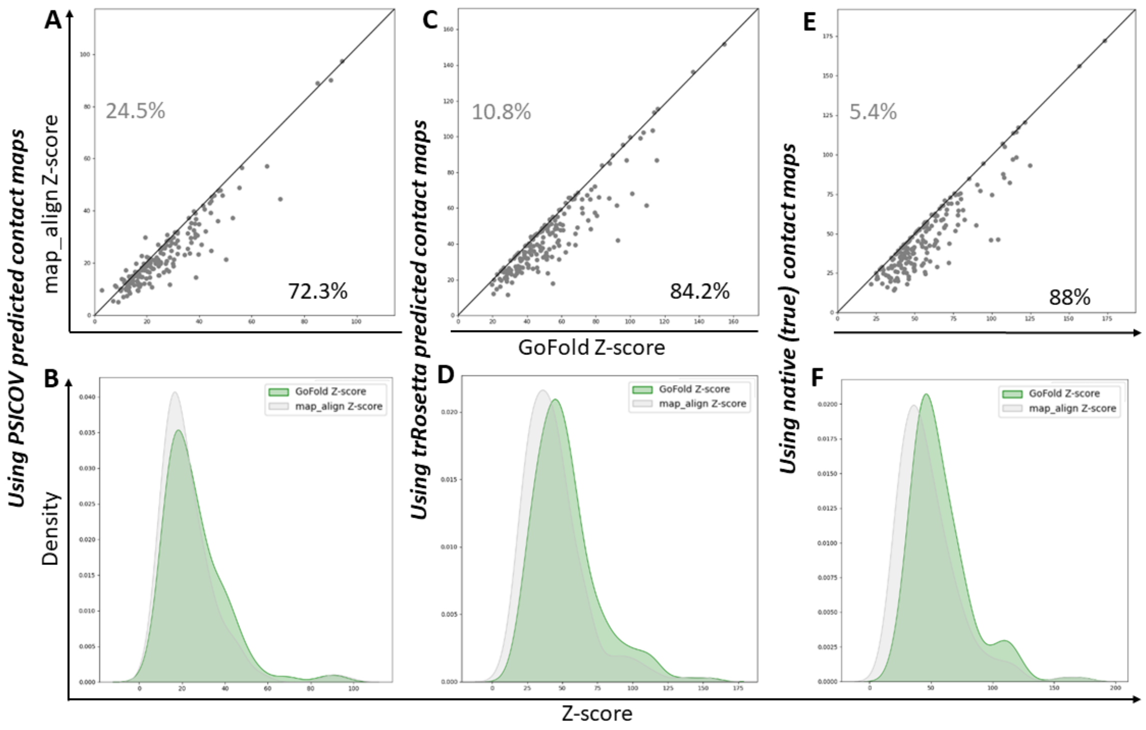

Supplementary Figure 2. A head-to-head Performance comparison of GoFold and map_align on PSICOV dataset based on the Z-score of predicted models. We include contact maps predicted by PSICOV, trRosetta, and true (native) contact maps. (A) GoFold versus map_align using PSICOV predicted contact maps, (B) Z-score distribution of models predicted by GoFold (in green) versus map_align (in grey) using PSICOV predicted contact maps, (C) GoFold versus map_align using trRosetta predicted contact maps, (D) Z-score distribution of models predicted by GoFold (in green) versus map_align (in grey) using trRosetta predicted contact maps, (E) GoFold versus map_align using native (or true) contact maps, (F) Z-score distribution of models predicted by GoFold (in green) versus map_align (in grey) using native (or true) contact maps.

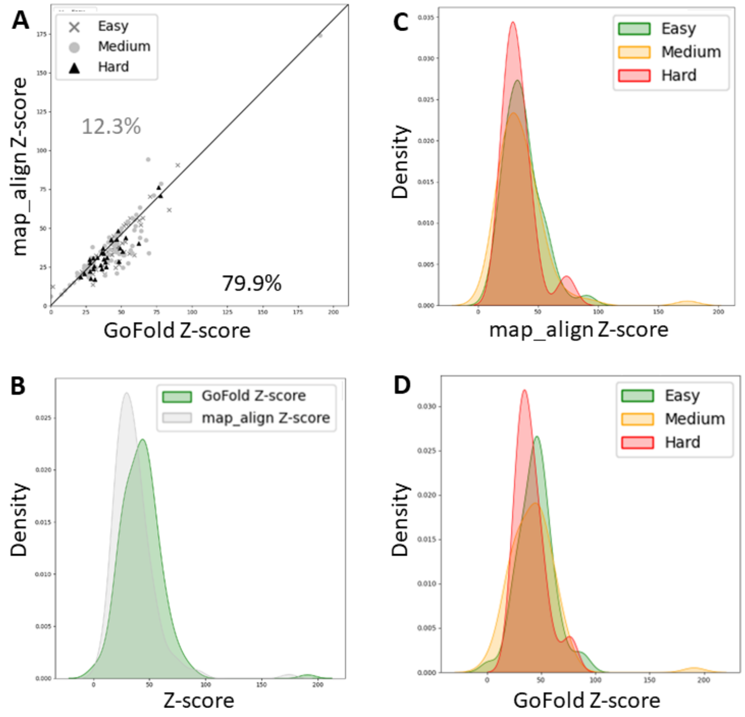

Supplementary Figure 2. A head-to-head Performance comparison of GoFold and map_align on CAMEO dataset based on the Z-score of predicted models. The target category is officially released by CAMEO. We include contact maps predicted by trRosetta for both GoFold and map_align. (A) GoFold versus map_align, (B) Z-score distribution of models predicted by GoFold (in green) versus map_align (in grey) over all targets, (C) Z-score distribution of models predicted by map_align over easy (in green), medium (in yellow), hard (in red) targets. (D) Z-score distribution of models predicted by GoFold over easy (in green), medium (in yellow), hard (in red) targets.
