# Supplementary material for: An interactive visualization tool for educational outreach in protein contact map overlap analysis
Source: Front Bioinform. 2024 Mar 15;4:1358550. doi: 10.3389/fbinf.2024.1358550 (PMC10982686; doi:10.3389/fbinf.2024.1358550)
Supplement: Supplementary file 2 [file DataSheet1.PDF]

GoFold Manual

Kevan Baker, Nathaniel Hughes, Sutanu Bhattacharya

December 7, 2023

Contents

1 Introduction **2**
1.1 Foreword . . . 2
1.2 GoFold - An Educational Tool . . . 2

2 Running & Licenses **3**
2.1 Running GoFold . . . 3
2.2 Licenses . . . 3

3 Modes **4**
3.1 3D Structure Matching . . . 4
3.2 2D Contact Matching . . . 5
3.3 Processing modes . . . 6
3.3.1 Batch .pdb processing . . . 6
3.3.2 Batch .pdb and .rr processing . . . 6

4 Acknowledgments **8**

1 Introduction

1.1 Foreword

As a computer scientist, I have had much to learn myself about the protein 3D structure prediction problem. While I have been in the process of learning for quite some time as a student, learning bioinformatics and this field as a computer science student has been very humbling. In effect, I have tried my best to build a software that can be useful to a humble beginner, such as I, who could have a genuine learning experience about the protein 3D structure prediction problem.

I find the fields of bioinformatics, computational biology, and protein folding to be noble and righteous in their line of work and the causes pursued. Thus, I'll be very happy and proud if even one person comes away from GoFold inspired to join into the field and contribute to the research that is being done on topics such as cancer and Alzheimer's.

Thank you for reading, Kevan Baker

1.2 GoFold - An Educational Tool

GoFold is an educational tool meant for people of all ages and backgrounds to try. The goal of the software is to get the user acquainted with the advanced concepts of protein 3D structure prediction. It has the goal of walking the user through simple, guided games that allow the user to both participate and learn about the protein 3D structure prediction problem. In addition, GoFold supports advanced modes for users who are more versed in the topic to analyze contact maps.

The name GoFold comes from the Godot Engine that GoFold is made in, and the protein folding problem. However, it also is meant to be an encouragement!

2 Running & Licenses

2.1 Running GoFold

GoFold is distributed as a binary, with an extra file that is used as a library for the binary to access resources that are part of the game. As such, the user need not install any other software to run GoFold, they must simply obtain the binary and its included library or folder.

Once the binary is obtained, it may be placed anywhere on the drive, along with the libraries or folders that are included with GoFold, and the user can execute the program from the GoFold binary.

2.2 Licenses

At this time, the GoFold source code is kept closed, with the goal of releasing the source code under the GPL at a later date.

The binary is released without liability or warranty from its distributors.

3 Modes

The various modes of GoFold can be accessed from the main menu, which greets the user upon start-up. In addition, the user can access the main menu from any of the modes by using the `Esc` key on the keyboard, and selecting "Return to Main Menu". From the same pause menu, the user can also access a "Help" button, which brings up a menu on the screen to tell the user about the mode they're currently in and details on how to use the mode.

GoFold currently exists of 2 basic modes, and 2 advanced modes. The basic modes are introduced to the user in a series of levels, like a game. The user proceeds through 3 levels, and is presented with their score at the end.

3.1 3D Structure Matching

This first mode is available from the main menu by pressing the "Start Structure Matching" button. From here, the user is greeted with a short text regarding the mode and the user's goal. To proceed through the mode, the user navigates using the "Next" button.

In the initial state, the game presents the user with a help overlay. In addition, the user can access help by pressing the `Esc` key, and selecting the "Help" option. The "Help" option reminds the user their goal and how to control the game. The help overlay details and points to what the user can interact with and how.

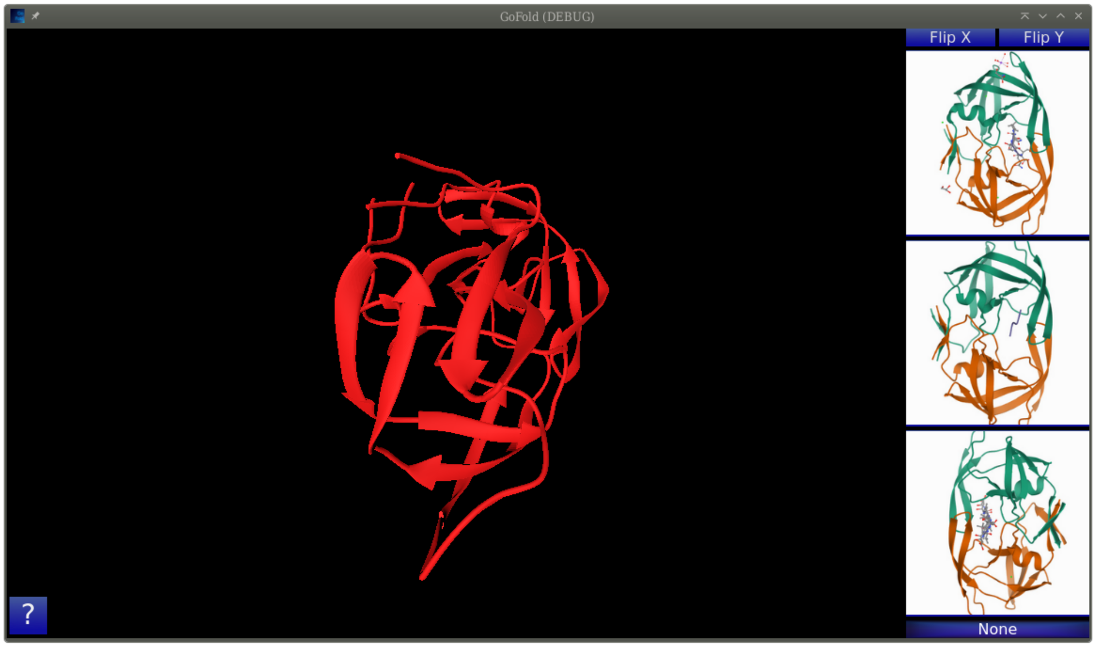

The goal of the user in this mode is to select from 3 given templates the closest template to their given target. The user's target is given in red, while the three templates are selectable from the right-hand side of the screen. When the user selects a template, they are able to see the template overlayed across the target in white. When the user is happy with a template, they are able to move to the next level with the "Next" button. The "Next" button remains hidden until the user has made a choice.

When the user has proceeded through each level, they are presented with their accuracy of choice between the templates and targets. The best possible templates for each target will achieve a 100%

score, while selecting less-than-optimal templates for the targets will lower the score percentage. Additionally, users will be presented with the total time taken to finish, however this time does *not* factor into the score. From this screen, the user is able to retry this mode to attempt a better score and see more information about their score and how it is calculated.

3.2 2D Contact Matching

The next mode of GoFold, Contact Matching, can be started from the main menu by pressing the "Start Contact Matching" button. From here, the user is greeted with a short text regarding the mode and the user's goal. To proceed through the mode, the user navigates using the "Next" button.

In the initial state, the game presents the user with a help overlay. In addition, the user can access help by pressing the `Esc` key, and selecting the "Help" option. The "Help" option reminds the user their goal and how to control the game. The help overlay details and points to what the user can interact with and how.

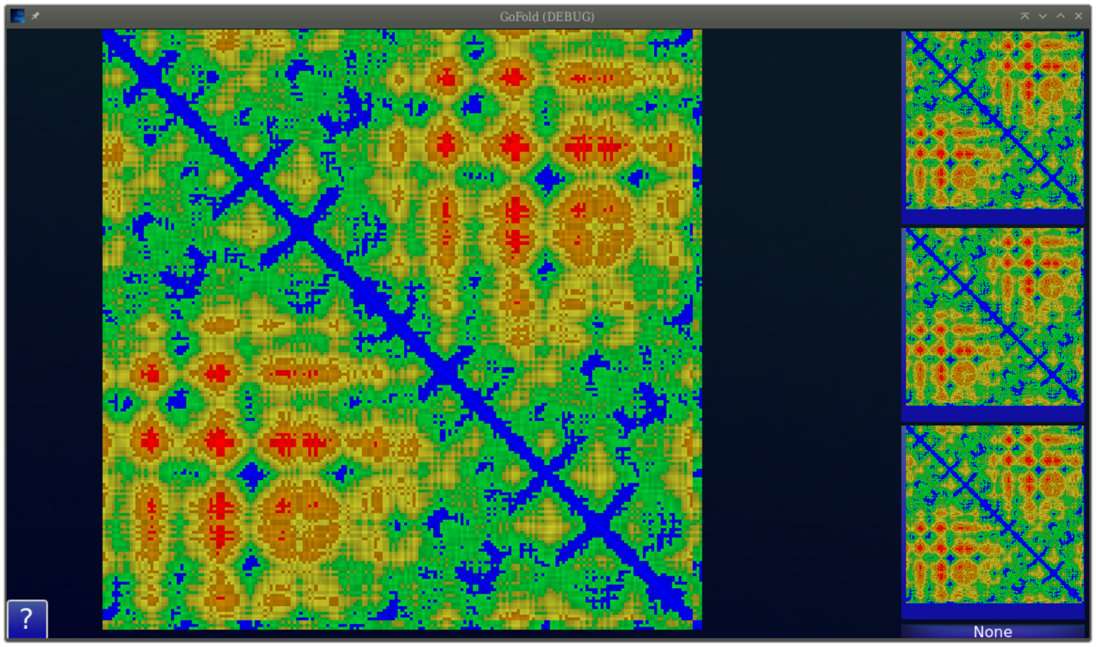

The user is presented with a large distance map, and three smaller distance maps on the right-hand side of the screen. The largest of the distance maps represents the user's target protein. The smaller three distance maps are template proteins for the user. The goal of the user is to select from the three templates which they think will be the best-fit template for their given target. When the user selects a template, the template and target's contact map overlap (CMO) is displayed. The target is displayed in grayscale, while the template's overlap is displayed in blue. Selecting the "None" option will return the target to its default state. When the user is happy with their choice of template, they can proceed to the next level with the "Next" button. The user will not be able to proceed to the next level until they have selected a template.

When the user has proceeded through each level, they are presented with their accuracy of choice between the templates and targets. The best possible templates for each target will achieve a 100% score, while selecting less-than-optimal templates for the targets will lower the score percentage. Additionally, users will be presented with the total time taken to finish, however this time does *not*

factor into the score. From this screen, the user is able to retry this mode to attempt a better score and see more information about their score and how it is calculated.

3.3 Processing modes

3.3.1 Batch .pdb processing

In this mode, the user is able to process alignment between pairs of proteins that are both in the PDB format. The way to facilitate this is to have the desired proteins within a folder. Then, the pairs are described in a "pairs.in" file, where each line in the file is the target name followed by the template name and a space. The last line in the file should not have a newline character at the end. In other words, there should be no empty lines in the file, including at the end of the file. The "pairs.in" file must be within the same folder at the same directory level as the proteins.

```
DirAccess loaded OK
Located pairs.in
Beginning iteration of proteins...
(There may be no output for a moment while GoFold works...)
1vfy amino sequence: DWIDSDACMICSKKFSLLNRKHHCRSCGGVFCQEHSSNSIPLPDLGIYEPVRVCDSCFEDYEFIVTD
2yw8 amino sequence: KDDEATHCRQCEKEFSISRRKHHCRNCGHIFCNTCSSNELALPSYPKPVRVCDSCHTLLLQ
1:1,2:2,3:3,4:4,5:5,6:6,7:7,8:8,9:9,10:10,11:11,12:12,13:13,14:14,15:15,16:16,17:17,18:18,19:19,20:20,21:21,22:22,23:23,24:24,25:25,26:26,
27:27,28:28,29:29,30:30,31:31,32:32,33:33,34:34,35:35,36:36,37:37,38:38,39:39,40:40,41:41,42:42,44:43,45:44,47:45,48:46,49:47,50:48,51
:49,52:50,53:51,54:52,55:53,56:54,57:55,58:56,59:57,60:58,61:59
Processed target: 1vfy template: 2yw8
Outputting files...
```

In this figure, the folder contains "1vfy.pdb", "2yw8.pdb", and "pairs.in". The pairs.in file simply contains "1vfy 2yw8", without any new line characters or carriage returns. However, if one desires to process many protein target-template pairs, they can do that as well by separating each target template pair with a newline character. But the user must beware not to use any empty lines, including at the end of the file.

Once the user starts this mode, they are presented with a button to open a folder. If the selection of the folder goes wrong, the user will be presented with a blank screen. The user may simply use the `Esc` key to return to the main menu, and then proceed back to this mode to try again. When the user selects a suitable candidate folder, GoFold will both print to the screen and output to files the results of the alignment between each target-template pair. The output will include the alignment, the proteins' fasta sequences, and files containing the contact maps of each individual protein. Additionally, a pairs.out file is generated that will be identical to the pairs.in, to confirm a successful run. Users are advised that GoFold will overwrite files that it writes out to. This includes each protein's .fasta, .map, and .map1 files. Additionally, the output is stored in "target-template.gf.aln".

3.3.2 Batch .pdb and .rr processing

In this mode, the user is able to process alignment between pairs of proteins where the target protein is in CASP residue-residue format and the template is in PDB format. The way to facilitate this is to have the desired proteins within a folder. Then, the pairs are described in a "pairs.in" file, where each line in the file is the target name followed by the template name and a space. The last line in the file should not have a newline character at the end. In other words, there should be no empty lines in the file, including at the end of the file. The "pairs.in" file must be within the same folder at the same directory level as the proteins.

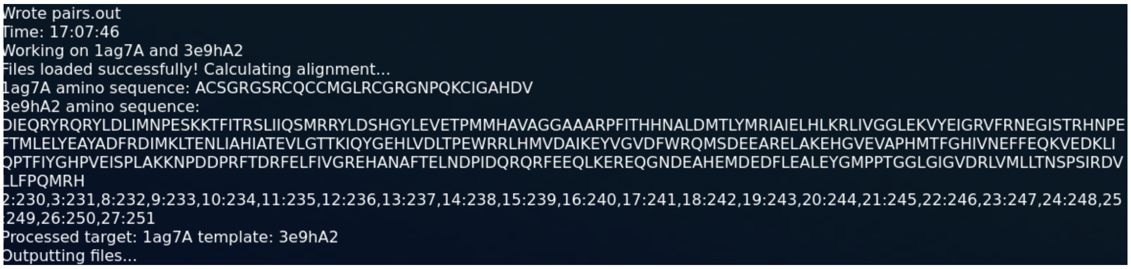

In this figure, the batch processing is done on a folder containing a single target-template pair, with 1ag7A as the target, and 3e9hA2 as the template. The amino acid fasta sequences are displayed, as well as the calculated alignment. The "pairs.in" file simply contains "1ag7A 3e9hA2", without the file extensions or any newline characters. However, if the user were to want more than one target-template pair, they would simply include those files as well in the same folder, and have them separated inside of pairs.in with a newline character.

This mode outputs to files as well, overwriting any existing files that may be in the place of where GoFold writes to. The output files are .fasta files for both the target and template, .gf.aln files for each target-template pair's alignment, a pairs.out file identical to pairs.in to confirm a successful run, and .map and .map1 files for each protein.

To successfully run this mode, the user must start the mode, and select a folder which satisfies the above conditions, namely having the targets, templates, and pairs.in all in the same folder, and the targets are in .rr extension format and the templates in .pdb extension format, with the pairs.in file constructed as detailed above. If the user is unable to successfully try the mode or wishes to rerun it, they must simply exit to the main menu via the `Esc` key's pause menu, and restart the mode.

4 Acknowledgments

Thank you to my advisor, Dr. Sutanu Bhattacarya. Dr. Bhattacharya has been a great inspiration to me in my career as a Master's student, and inspired me to go on to become a Ph.D student. Thank you to Nathaniel Hughes as well, for greatly helping me with the GoFold project and putting in a lot of effort. I am proud of the work we have accomplished.
